# Differences by race in the associations between neighborhood crime and violence and glycemic control among adults with type 2 diabetes

**DOI:** 10.1371/journal.pone.0279234

**Published:** 2022-12-15

**Authors:** Olaitan Akinboboye, Joni S. Williams, Oluwatoyin Olukotun, Leonard E. Egede

**Affiliations:** 1 Department of Public and Community Health, Medical College of Wisconsin, Milwaukee, WI, United States of America; 2 Division of General Internal Medicine, Department of Medicine, Froedtert & The Medical College of Wisconsin, Milwaukee, WI, United States of America; 3 Center for Advancing Population Science (CAPS), Medical College of Wisconsin, Milwaukee, WI, United States of America; 4 School of Nursing, University of Portland, Portland, OR, United States of America; Iuliu Hațieganu University of Medicine and Pharmacy: Universitatea de Medicina si Farmacie Iuliu Hatieganu, ROMANIA

## Abstract

**Background:**

Limited data exist on the differential association between neighborhood characteristics such as crime and violence and diabetes outcomes by race.

**Objective:**

To examine racial differences in the relationship between neighborhood characteristics (crime and violence) and glycemic control in a sample of adults with type 2 diabetes (T2DM).

**Design:**

A cross-sectional study.

**Participants:**

601 adults with T2DM from the Southeastern United States.

**Measurements:**

Outcome was glycemic control. Neighborhood violence and crime were the primary independent variable, and previously validated scales and indices were used to assess neighborhood crime and violence. Covariates included age, gender, education, marital status, income, hours of work per week, duration of diabetes, comorbidity, health status, and site of recruitment. Multiple linear regression was used to assess the relationship between neighborhood characteristics (violence and crime) and glycemic control adjusting for relevant covariates.

**Results:**

Approximately 66% of the sample population was Black with ages ranging between 49–71 years. The unadjusted mean hemoglobin A1c (HbA1c) was significantly higher for Black adults compared to White adults (8.0 ± 2.0 vs. 7.8 ± 1.6; p = 0.002). In the fully adjusted stratified model, glycemic control was significantly associated with neighborhood crime (β-coefficient: 0.36; 95% CI 0.07, 0.65) and neighborhood violence (β-coefficient: 0.14; 95% CI 0.003, 0.28) for White adults in the fully adjusted model; these relationships were not significant for Black adults.

**Conclusion:**

In this sample of adults with T2DM, neighborhood crime and violence were significantly associated with glycemic control for White adults, but not for Black adults. Additional research is needed to understand perceptions of neighborhood crime and violence between White adults and Black adults with T2DM.

## Introduction

More than 34 million people, or approximately 10.5% of the United States population lives with diabetes, making it one of the most prevalent and costly chronic diseases, with an associated expenditure of more than $327 billion per annum [1, 2]. The prevalence of diabetes among Black adults is 11.7% compared to 7.5% for White adults [1, 2]. Existing literature has shown Black adults with diabetes have poorer outcomes such as being 2.3 times more likely to die from diabetes compared to White adults and having a higher risk for adverse complications such as lower extremity amputations and chronic kidney disease [3, 4]. While socioeconomic status (SES), health insurance status, and access to health care have been identified as important contributors to disparities in diabetes outcomes for Black adults, much of the observed differences in glycemic control among adults living with diabetes remains unexplained by these factors alone [5, 6] implying other contextual factors including socio-environments, defined as immediate physical surroundings and social relationships within a given environment such as neighborhoods, may account for these disparities [7–12].

Social determinants of health (SDOH) are the conditions in the environment in which people are born, grow, work, live, and age which influences health outcomes [13]. SDOH are factors from the neighborhood that influence health outcomes of communities and people [14]. Brown and colleagues postulated a framework that evaluates the relationship between components of socio-economic position which include neighborhood characteristics and health outcomes in individuals with type 2 diabetes [7]. The framework hypothesizes that socio-economic position such as neighborhood characteristics impact type 2 diabetes health outcomes [7]. This hypothesis has been supported by studies, which have shown that neighborhood characteristics such as neighborhood crime and violence, lack of social amenities, poor social support, and resilience have served as barriers to self-care and glycemic control in adults living with diabetes [15–21]. Evidence shows individuals with perceptions of high levels of crime and violence in their neighborhoods often report suboptimal health behaviors such as physical inactivity, which has led to higher rates of obesity, other comorbid conditions, and adverse complications in some communities [22, 23]. Furthermore, studies have shown that negative life experiences within neighborhoods underline racial differences in health outcomes [24–26], being associated with heightened threat-related emotional functioning, and psychophysiological and behavioral responses to those threats [27]. The latter is closely related to self-care behaviors, which has been shown to mediate the relationship between neighborhood characteristics and glycemic control [6, 7].

While studies have explored the relationship between neighborhood characteristics and glycemic control generally [10,12], evidence demonstrating the differential relationship between neighborhood characteristics such as crime and violence and glycemic control by race remains less well studied. Therefore, to address this gap, this study assesses racial differences in the relationship between neighborhood crime and violence and glycemic control in adults with diabetes living in the southeastern United States. Given racial differences in threat-related emotional function and self-care behavior responses to exposure to neighborhood violence and neighborhood crime, we hypothesize that an increase in neighborhood crime and violence is associated with poorer glycemic control and that there will be differences by race.

## Materials and methods

### Study population

This is a secondary analysis of data from 601 White and Black adults who were recruited from two adult primary care clinics in the southeastern United States between July 3, 2012 and September 30, 2013. Participants were recruited from two sites: The Medical University of South Carolina (MUSC) and the Ralph H. Johnson Veteran Affairs (VA) Medical Center. These sites were used for study recruitment because they served the population of interest and were the affiliated institutions of the research team. Prior to study enrollment, all procedures were approved by the Institutional Review Board at MUSC and the VA Research and Development Committee and complied to all ethical standards (PRO00017676). Individuals ages 18 years and older and proficient in English proficiency with a diagnosis of type 2 diabetes and a hemoglobin A1c (HbA1c) within the prior 6 months in their medical records were eligible to participate in the study. Individuals were ineligible to participate in the study if they were disoriented during the screening interview or were determined by validated screening instruments to have active psychosis, active mental disorder, or reported substance abuse/dependency.

### Data collection

Eligible participants were identified from the electronic clinic data by trained and approved program staff listed on the IRB application. Each eligible patient was subsequently approached in the clinic and provided a detailed description of the study. The response rate for this study was 90% among those who were approached for study participation. Patients eligible and interested were consented, and validated questionnaires assessing demographic information [28], perceptions of neighborhood crime and violence [29], and quality of life [30] were administered to each participant. In addition, program staff who consented and enrolled eligible patients in the study were required to conduct mock study visits with fellow program staff, and this ensured that the consent and study assessment process was standardized.

### Outcome measure

Hemoglobin A1c (HbA1c) was abstracted from electronic medical records using values observed within the previous 6 months before survey completion. For all study participants, the most recent HbA1c of record was abstracted and used for the analysis. HbA1c was analyzed as a continuous variable.

### Primary independent variables: Neighborhood crime and violence

Neighborhood crime and neighborhood violence were the independent variables of interest and were based on evidence from a prior validation study using six scales and four indices to assess neighborhood characteristics [29]. Four questions were used to assess neighborhood violence:”During the past six months, how often was there (1) a fight in this neighborhood in which a weapon was used?”; (2) “Any gang fights?”; (3) “A sexual assault or rape”; and (4) “A robbery or mugging”. Neighborhood violence response scale ranged from 1 (Often) to 4 (Never). The score for neighborhood violence ranged from 4 to 16 and was analyzed as a continuous variable, with higher numbers representing increased neighborhood violence [29]. The neighborhood violence scale (α = 0.83 to 0.85) has a high internal consistency, based on the Cronbach’s alpha range, and test-retest reliability (Confidence Interval: 0.64–0.87) [29]. Neighborhood crime was assessed using a single item prompt: “My neighborhood is safe from crime”, and responses ranged from not safe (1) to extremely safe (4). The neighborhood crime score ranged from 1 to 4 and was analyzed as a continuous variable [29]. Similar to the scale for neighborhood violence, higher numbers on the neighborhood crime scale represent increased neighborhood crime. The Cronbach-alpha for the neighborhood crime scale ranges from α = 0.77 to 0.82, and the test-retest reliability has a confidence interval of 0.67–0.88 [29].

### Covariates

Demographic variables included age, gender, race, marital status, education, and annual personal income [28]. Age and education were continuous variables. Race wase dichotomized as Black and White. Marital status was categorized as never married, married separated, and widowed. Annual personal income was categorized as <$9,999, $10,000-$14,999, $15,000-$19,999, $20,000-$24,999, $25,000-$34,999, $35,000-$49,999, $50,000-$74,999, and $75,000 and greater. Other covariates in the analysis included hours of work per week, duration of diabetes, comorbidity, health status, and recruitment site. Hours of work per week and duration of diabetes were continuous variables. Comorbidity was calculated using the Charlson comorbidity index and reported as a continuous variable [31]. Health status was assessed using the SF-12 and was categorized as excellent, very good, good, fair, and poor [30].

### Statistical analysis

Descriptive statistics were used to describe the data. First, we compared sample demographics using chi-square statistics for categorical variables and one-way analysis of variance (ANOVA) for continuous variable. Next, multiple linear regression was used to assess the relationship between neighborhood characteristics (crime and violence) and glycemic control, adjusting for relevant covariates in the overall sample. Then, consistent with the hypothesis, a stratified analysis was performed, and multiple linear regression was used to assess the relationship between neighborhood characteristics (crime and violence) and glycemic control, adjusting for relevant covariates in both White and Black adults. Variables were selected into the model based on two criteria: clinical relevance based on established literature and statistical relevance based on a significant bivariate relationship at a p-value less than 0.25. The outcome variable was glycemic control measured by HbA1c. Covariates included age, gender, race, marital status, education, annual personal income, hours of work per week, duration of diabetes, comorbidity, health status, and recruitment site. A two-tailed p-value of 0.05 was used to assess statistical significance. Statistical analysis was performed with STATA version 14.0.

## Results

Table 1 shows the demographic characteristics of the sample by race. Of the total sample, 203 were White, and 398 were Black. For White adults, the mean age was approximately 65 years; the mean educational attainment was 14.3 years; and the mean hours of work per week were nearly 10. In contrast, Black adults, were significantly younger (mean age = 59.9 years; p = 0.020), had less educational attainment (13 years; p<0.001), and worked more hours per week (approximately 14 hours; p = 0.019). White adults were predominantly male (77.3%), married (62.6%), had an income range of $35,000 to $49,999 (21.9%), and reported good health status (42.4%). Similarly, Black adults were also primarily male (53.1%) and married (42.4%); however, the majority had an income range of $0 to $9,999 (26.4%) and reported being in fair health (41.6%). There were statistically significant differences between Black and White adults with respect to educational attainment, age, hours of work per week, gender, marital status, and income.

**Table 1:** Sample demographics by race.

Variable	All	White	Black	P-value
Sample	601	203	398	--
**Age (years)**	61.5 ± 10.9	64.7 ± 9.6	59.9 ± 11.1	**0.020**
**Education (years)**	13.4 ± 2.8	14.3 ± 3.2	13.0 ± 2.6	**<0.001**
**Hours of work/week (hours)**	12.3 ± 18.9	9.7 ± 17.0	13.6 ± 19.7	**0.019**
**Duration of Diabetes (years)**	12.3 ± 9.2	12.9 ± 9.3	11.9 ± 9.1	0.732
**Charlson Comorbidity Score**	25.7 ± 2.2	25.9 ± 2.2	25.6 ± 2.3	0.408
**Gender**				**<0.001**
Male	61.3	77.3	53.1	
Female	38.7	22.7	46.9	
**Marital status**				**<0.001**
Never Married	11.3	6.9	13.6	
Married	49.2	62.6	42.4	
Separated	28.3	21.7	31.7	
Widowed	11.2	8.9	12.3	
**Income**				**<0.001**
$0 - $9,999	20.1	7.7	26.4	
$10,000 - $14,999	11.5	7.7	13.5	
$15,000 - $19,999	10.3	9.7	10.6	
$20,000 - $24,999	10.1	9.7	10.4	
$25,000 - $34,999	14.8	10.7	16.8	
$35,000 - $49,999	13.4	21.9	9.1	
$50,000 - $74,999	10.3	12.2	9.3	
$75,000 or more	9.4	20.4	3.9	
**Health Status**				0.171
Excellent	1.3	2.0	1.0	
Very Good	11.9	13.3	11.3	
Good	38.9	42.4	37.1	
Fair	38.2	31.5	41.6	
Poor	9.6	10.8	9.0	
**Site**				0.284
MUSC	51.8	48.8	53.4	
VAMC	48.2	51.2	46.6	

Bold text indicates significance at p-value<0.05. All numbers represent mean ± standard deviation or percentages. Abbreviations: MUSC, Medical University of South Carolina; VAMC, Veteran Affairs Medical Center.

Table 2 shows the relationship between neighborhood violence and neighborhood crime scores and HbA1c level by race. There were statistically significant differences in neighborhood violence scores and HbA1c levels between White and Black adults. Participants identifying as Black reported significantly higher neighborhood violence scores (5.3 ± 2.1 vs 4.9 ± 1.8; p = 0.040) and had higher HbA1c levels (8.0 ± 2.0 vs 7.8 ± 1.6; p = 0.002) compared to White adults. There was no significant difference in neighborhood crime between the groups (p = 0.489).

**Table 2:** Neighborhood violence, crime and glycemic control by race.

Neighborhood factors	All	White	Black	P-value
Neighborhood violence	5.1 ± 1.9	4.9 ± 1.8	5.3 ± 2.1	0.040*
Neighborhood crime	2.0 ± 0.8	1.9 ± 0.8	2.2 ± 0.8	0.489
HbA1c level	7.9 ± 1.8	7.8 ± 1.6	8.0 ± 2.0	0.002*

*Bold text indicates significance at P-value<0.05. All numbers represent mean ± standard deviation. Abbreviation: HbA1c = Hemoglobin A1c

Table 3 shows the fully adjusted model for the relationship between neighborhood crime and violence and glycemic control stratified by race. In the fully adjusted model, glycemic control was significantly associated with neighborhood crime (β = 0.36; 95% CI (0.07, 0.65)) and neighborhood violence (β = 0.14; 95% CI (0.003, 0.28)) for White adults. However, the relationships between neighborhood crime and violence were not significant for Black adults.

**Table 3:** Adjusted linear regression model of the relationship between neighborhood crime and neighborhood violence and glycemic control by race.

	White	Black
	HbA1c β-coefficient (95% Confidence Interval)	HbA1c β-coefficient (95% Confidence Interval)
**Neighborhood crime**	0.36 (0.07, 0.65) *	0.06 (-0.20, 0.32)
**Neighborhood violence**	0.14 (0.003, 0.28) *	0.23 (-0.08, 0.12)

Significance at ***p<0.001, **p<0.01, *p-value<0.05.

Dependent variable = HbA1c; Independent variables = neighborhood crime and violence.

Adjusted for: age, sex, marital status, duration of diabetes, education, hours worked per week, income, health status, comorbidity, site.

## Discussion

In this cross-sectional study of Black and White adults with diabetes from the southeastern United States, we found that neighborhood crime and violence were significantly associated with poor glycemic control among White adult, but not among Black adults, after adjusting for relevant covariates. In contrast, in the full sample without stratification, there were no significant relationships between neighborhood crime and violence and glycemic control in adults with type 2 diabetes after full adjustment. These findings suggest race influences the relationship between neighborhood crime and violence and glycemic control in adults with type 2 diabetes.

In our study, neighborhood crime and neighborhood violence were significantly associated with poorer glycemic control in White adults, but not in Black adults. This is a novel finding as most evidence has shown neighborhood crime and neighborhood violence to be associated with negative health outcomes in racial and ethnic minorities [27, 32]. It is noteworthy to emphasize, however, that consistent with previous studies [33, 34] Black adults in this study reported a higher average exposure to neighborhood violence and neighborhood crime compared to White adults in the study, despite no significant relationships observed.

The racial differences in the relationship between neighborhood crime and neighborhood violence and glycemic control in this study could be due to the difference in psychophysiological responses to threats between White and Black adults [25]. White adults demonstrate an increased autonomic response to exposures to threats compared to Black adults [25]. In addition, studies have shown that Black adults exhibit high levels of resiliency despite increased exposures to adverse social determinants of health such as neighborhood crime and violence [35–37]. Furthermore, Black adults have been shown to adapt to stressors [38], where some possess inherent qualities that make them resilient, while others grow and develop in environments that teach them how to be resilient [39]. Psychosocial and coping factors such as resilience may have moderated the effect of neighborhood violence and neighborhood crime on glycemic control in Black adults; however, these factors were not accounted for in the analysis.

Structural policies that encourage structural racism and residential segregation have forced Black adults to live in disadvantaged neighborhoods with chronic exposure to neighborhood violence and crime, which might have resulted in the development of adaptations by Black adults to cope with these stressors [38, 39]. This might not be the case for White adults who are able to reside in the most resourceful and safe neighborhoods [40]. Future studies should further explain the mechanisms and factors underlying the racial differences observed in this study.

The major strength of this study is our large sample size, and the use of validated measures of neighborhood characteristics; however, the study has some limitations worth acknowledging. First, this study analyzed cross-sectional data, which makes it difficult to ascertain causality. We cannot determine with certainty that neighborhoods with high violence and crime are the causes of poor glycemic control or if adults with poor glycemic control have high rates of crime and violence in their neighborhoods. Second, there were confounding factors not accounted for in the analysis. Based on available data, for example, we were not able to assess the length of time adults lived in their respective neighborhoods for which they were reporting or for additional clinical factors that may have influenced the relationship between neighborhood characteristics and glycemic control. Third, the study population was a sample from the southeastern United States and limited only to Black and White adults; therefore, findings may not be generalizable to individuals with diabetes from other regions of the country or to other racial or ethnic populations. Future studies should use a nationally representative dataset to assess this relationship.

## Conclusions

In this cross-sectional study of adults with type 2 diabetes from southeastern United States stratified by race and adjusted for relevant covariates, neighborhood crime and neighborhood violence were significantly associated with glycemic control in White adults, but not in Black adults. Additional research is needed to understand whether neighborhood crime and violence are perceived differently between White and Black adults with type 2 diabetes.
